# Characterization of Mutations in the Mitochondrial Encoded Electron Transport Chain Complexes in Acute Myeloid Leukemia

**DOI:** 10.1038/s41598-018-31489-0

**Published:** 2018-09-05

**Authors:** Sharon Wu, Mojtaba Akhtari, Houda Alachkar

**Affiliations:** 1 USC School of Pharmacy, University of Southern California, Los Angeles, CA USA; 2 Norris Comprehensive Cancer Center, University of Southern California, Los Angeles, CA USA

## Abstract

Acute Myeloid Leukemia is a devastating and heterogeneous, hematological malignancy characterized by the uncontrolled proliferation of undifferentiated myeloid progenitor cells—blasts. Mutations in certain mitochondrial proteins, such as IDH2 have been shown to contribute to leukemogenesis. However, the role of mutations in mitochondrial-encoded Electron Transport Chain (ETC) genes have thus far not been well elucidated in AML. Here, we use TCGA data to characterize mutations in the ETC genes and their association with clinical outcomes in AML. We found that mitochondrial ETC mutations—in Complex I, III, IV and/or V (ATP Synthase)—were present in 8% of patients with AML and were significantly more frequent in older patients. Patients with ETC mutations had worse overall survival than ETC wild type patients (OS: 9.3 vs 20.1 months; p-value: 0.007). Additionally, mutations in either or both Complex I and IV were associated with *TP53* mutations (p-value: 0.009), and among *TP53* mutated patients, mutations in either or both Complex I and IV were significantly associated with worse overall survival (OS: 0.85 vs 9.4 months; p-value: 0.008). Elucidation of the mechanisms by which ETC mutations contribute to AML pathogenesis and progression would facilitate the development of novel therapeutic targets.

## Introduction

Oxidative phosphorylation (OXPHOS) is a key intracellular process that generates ATP to power the cell. It is the last step in cellular respiration whereby cofactors, such as NADH and FADH_2_, generated in previous steps in respiration donate electrons to the Electron Transport Chain (ETC) to generate a H^+^ gradient that drives ATP synthase to convert ADP to ATP^1^. The ETC comprises of four complexes and an ATP synthase and is located on the inner membrane of the mitochondria^1^. During OXPHOS, however, some electrons may leak from the different electron transport chain complexes and bind with molecular oxygen to form superoxide anions. Superoxide anions bind to iron-sulfur cores of important cellular enzymes and inactivate them, causing shut down of pivotal intracellular processes^2,3^. Superoxide anions are detoxified by an important mitochondrial antioxidant called SOD2, which converts superoxide anions to H_2_O_2_. H_2_O_2_ is further detoxified by glutathione peroxidase 1 (GPX1). H_2_O_2_ may also interact with Fe^2+^ to generate OH^−^, or hydroxyl radicals^4,5^. Thus, mutations or defects in complexes of the ETC could have significant effects on the metabolic balance of the mitochondria.

OXPHOS downregulation resulting from a decrease in mtDNA content was reported in several malignancies including breast cancer, gastric cancer, hepatocellular carcinoma and non–small cell lung cancer (NSCLC), and was found to be associated with poor clinical outcome and correlated with invasive and metastatic tumors^6,7^. On the other hand, recent studies suggest that particular cancers such as acute lymphoblastic leukemia (ALL), non-Hodgkin lymphoma, prostate cancer, head and neck cancer, and thyroid cancer among others exhibit increased mtDNA content and these malignant cells rely heavily on OXPHOS for their energy source and survival^8^. Mitochondrial DNA (mtDNA) encodes 13 subunits of the ETC complexes: I, III, IV and V (ATP Synthase)^9^. Mutations in mtDNA have been identified in several malignancies^10^. Mitochondrial mutations were found to be important drivers in prostate cancer aggression^11^ and may contribute to hepatocellular carcinoma and colorectal cancer progression^10,12^.

Acute Myeloid Leukemia (AML) is a devastating and heterogeneous hematological malignancy characterized by the uncontrolled proliferation of undifferentiated myeloid progenitor cells—known as blasts^13^. The altered metabolic state of AML cells and the role of the mitochondria has been shown to contribute to its pathogenesis^14–16^. Mutations in the mitochondrial enzyme isocitrate dehydrogenase-2 (IDH2) have been identified as oncogenic drivers of AML. They convert alpha-ketoglutarate into the R-enantiomer of 2-hydroxyglutarate, which is associated with DNA hypermethylation, epigenetic changes, reduced ATP synthase activity and overall reduced mitochondrial energy metabolism^17–19^.

While mitochondrial DNA mutations have been previously reported in hematologic malignancies including AML^10,20^, mutations in the ETC genes encoded by the mitochondria have not been fully characterized in AML. Thus, we used data of patients with AML from the Cancer Genome Atlas (TCGA) to investigate mitochondrial ETC mutations in AML and assessed their association with clinical and molecular characteristics and with patients’ clinical outcome.

## Results

### Frequency of ETC complex gene mutations

Of the 200 patients included in this analysis, 16 (8%) patients had a mutation in one or more of the mitochondrial encoded ETC complexes genes. Each mitochondrial encoded ETC gene codes a different subunit of each complex. Four patients had mutations in Complex I. Complex I has seven mitochondrial encoded genes-*MT-ND1*, *MT-ND2*, *MT-ND3*, *MT-ND4*, *MT-ND4L*, *MT-ND5* and *MT-ND6*—where one patient has a missense mutation in *MT-ND1* and *MT-ND5*, two patients have missense mutations in *MT-ND5* and one patient has a truncating mutation in *ND5*. Three patients had missense mutations in the mitochondrial encoded *MT-CYB* gene in Complex III. Complex IV has three mitochondrial encoded genes—*MT*-*CO1*, *MT-CO2* and *MT-CO3*. Two patients had missense mutations in *MT-CO1*, five patients had missense mutations in *MT-CO2* and two patients had missense mutations in *MT-CO3*. One patient had a missense mutation in *MT-ATP6* of the ATP Synthase; but no patients had mutations in *MT-ATP8*. One patient had a mutation in both Complex I and Complex IV. Because Complex II is composed of four nuclear gene encoded subunits, mutations in Complex II were not included in this study. Frequencies of mutations are summarized in Table 1

**Table 1:** Frequency of Mutations in the mtDNA ETC genes in Patients with AML (^+^denotes one patient with mutations in both *ND1* and *ND5*).

Complex	Gene	Amino Acid Change	Mutation Type	No. of Patients
Complex I	*ND1*	Y43H^+^	Missense	1 (0.5%)
	*ND2*	—	—	—
	*ND3*	—	—	—
	*ND4*	—	—	—
	*ND4L*	—	—	—
	*ND5*	S345P, S476P	Missense	4 (2.0%)
		L260P^+^	Missense	
		N452S	Missense	
		P242Qfs*?	Truncating	
	*ND6*	—	—	—
Complex III	*CYB*	V73M	Missense	3 (1.50%)
		M89I	Missense	
		A302T	Missense	
Complex IV	*CO1*	G435E	Missense	2 (1.0%)
		M237V	Missense	
	*CO2*	L126S	Missense	5 (2.5%)
		D173N	Missense	
		V191M	Missense	
		T66A	Missense	
		D112N	Missense	
	*CO3*	L175P	Missense	2 (1.0%)
		V254A	Missense	
ATP Synthase	*ATP6*	M60T	Missense	1 (0.5%)
	*ATP8*	—	—	—

.

### Association of ETC complex gene mutations with clinical characteristics

We assessed the association between ETC gene mutations and primary patient characteristics. The results are summarized in Table 2. No significant difference in median white blood cell count, peripheral blood blasts and bone marrow blasts were detected between patients with mutations and patients without mutations. Mutations in mitochondrial DNA of any of the ETC genes were significantly associated with age (median: 68.5 vs 57; p-value: 0.001), the M0 FAB classification of AML (%: 26.7 vs 8.2; p-value: 0.04) and poor molecular risk (%: 50 vs 23.4; p-value: 0.035).

**Table 2:** Clinical characteristics of patients with AML according to mutational status of mitochondrial ETC genes.

Characteristic	No Mutations (n = 184)	Mutations (n = 16)	*p-value*
**Age, median** (years)	57	68.5	0.001
*Young* (<60)	106 (57.6%)	3 (18.8%)	0.003
*Old* (≥*60)*	78 (42.4%)	13 (81.2%)	
**Sex**			0.30
Female (*n, %*)	87 (47.3%)	5 (31.3%)	
Male (*n, %*)	97 (52.7%)	11 (68.7%)	
**FAB**			
M0 (n, %)	15 (8.2%)	4 (26.7%)	0.04
M1 (*n, %*)	41 (22.4%)	5 (33.3%)	0.35
M2 *(n, %*)	41 (22.4%)	3 (20%)	>0.99
M3 (*n, %*)	19 (10.3%)	1 (6.25%)	>0.99
M4 (*n, %*)	39 (21.3%)	2 (13.3%)	0.74
M5 *(n, %*)	22 (12.0%)	0	0.38
M6 (*n, %*)	3 (1.64%)	0	>0.99
M7 (*n, %*)	3 (1.64%)	0	>0.99
**WB Count, median**	15.6	21.5	0.79
*ln* (*WB Count*)	2.60	2.74	0.75
**% BM Blast, median**	72	77	0.69
**% PB Blast, median**	34.5	49	0.99
**Risk Status**			
*Poor* (*n, %*)	43 (23.4%)	8 (50.0%)	0.035
*Intermediate* (*n, %*)	99 (53.8%)	7 (43.8%)	0.44
*Good* (*n, %*)	38 (20.7%)	1 (6.25%)	0.20
**Cytogenetic Status**			0.80
*Normal* (*n, %*)	85 (47.5%)	7 (43.8%)	
*Abnormal* (*n, %*)	94 (52.5%)	9 (62.5%)	
**Transplant (Y/N)**			0.79
*No* (*n, %*)	102 (55.4%)	10 (62.5%)	
*Yes (n, %*)	82 (44.6%)	6 (37.5%)	

P-values calculated using non-parametric Mann-Whitney U or Fisher’s Exact test.

Next, we stratified the analysis by ETC complex to see if mutations in a specific complex were associated with patient’s clinical characteristics. Only Complex I, III and IV had enough patients with mutations to be included in the analysis, n = 4, n = 3 and n = 9, respectively. Patients were also grouped together by the presence of one or more mutations in either or both Complex I and IV (n = 12). Mutations in Complex I, alone, or in Complex III, alone, were not significantly associated with any clinical characteristics (Supplemental Tables S1 and S2. Mutations in Complex IV, alone, were significantly associated with age (median: 72 vs 57; p-value: 0.009) and the M0 FAB sub-classification of AML (%: 33.3 vs 8.47; p-value: 0.04; Supplemental Table S3). Mutations in Complex I and IV, either or both, were significantly associated with age (median: 71.5 vs 57; p-value: 0.008) and poor molecular risk (%: 58.3 vs 23.4; p-value: 0.015; Supplemental Table S4).

### Association of ETC complex gene mutations with molecular characteristics

**Figure 1:**
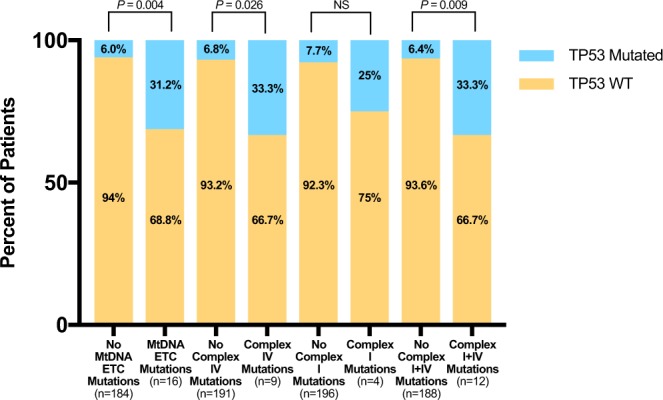
Distribution of *TP53* in AML patients stratified by mtDNA ETC gene mutations. The percentage of patients with different types of mtDNA mutations that have *TP53* mutations. Fisher Exact test was used for statistical analysis to determine if there was a statistically significant difference in proportion of patients with *TP53* mutations between patients with or without mtDNA ETC mutations.

Patients with mutations in the ETC complex genes were more frequently mutated with *TP53* (%: 31.3 vs 5.98; p-value: 0.004; Table 3). Mutations in Complex IV, alone, were also associated with mutations in *TP53* (%: 33.3 vs 6.81; p-value: 0.026; Supplemental Table S5). When combined, either or both Complex I and IV mutations remained associated with *TP53* mutation (%: 33.3 vs 6.38; p-value: 0.009; Supplemental Table S6; Fig. 1). No association was found between mutations in the ETC genes, Complex I alone or Complex III alone, and other mutations reported in AML (Supplemental Tables S7 and S8.

**Table 3:** Molecular characteristics of patients with AML according to mutational status of mitochondrial ETC genes. P-values calculated using Fisher’s Exact test.

Gene	No Mutations (n = 184)	Mutations (n = 16)	*p-value*
*FLT3*	53 (8.8%)	3 (18.8%)	0.56
*TP53*	11 (5.98%)	5 (31.3%)	0.004
*DNMT3A*	45 (24.5%)	4 (25%)	>0.99
*CEBPA*	11 (5.98%)	2 (12.5%)	0.28
*NRAS*	15 (8.15%)	0	0.62
*TET2*	16 (8.70%)	1 (6.25%)	>0.99
*IDH1*	18 (9.78%)	1 (6.25%)	>0.99
*IDH2*	18 (9.78%)	2 (12.5%)	0.67
*RUNX1*	16 (8.70%)	1 (6.25%)	>0.99
*NPM1*	51 (27.7%)	3 (18.8%)	0.57
*WT1*	12 (6.52%)	0	0.60

### Association of mtDNA ETC gene mutations with clinical outcome

**Figure 2:**
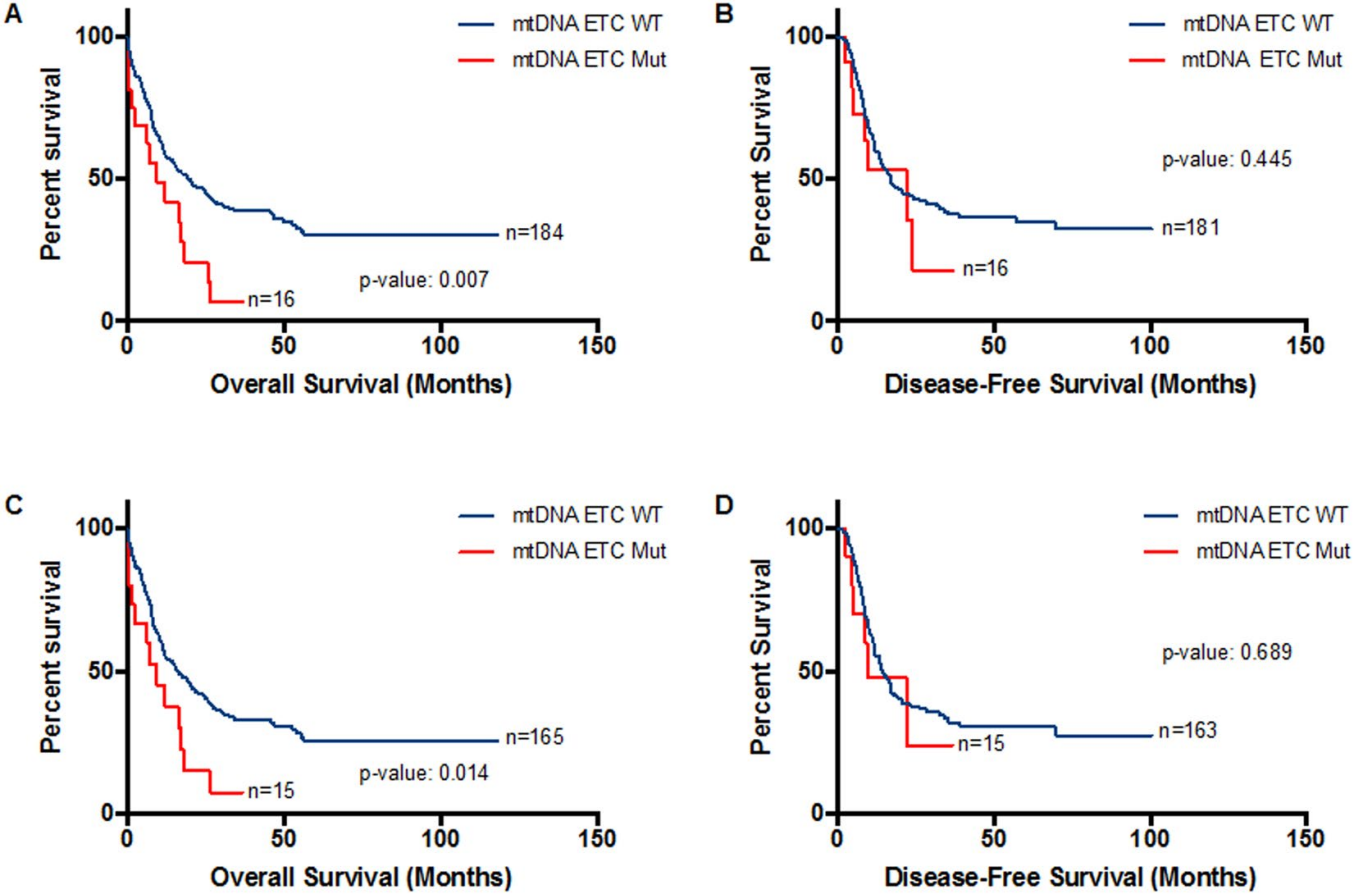
Association of ETC complex gene mutations with clinical outcome. Kaplan-Meier survival curves. (**A**) Overall Survival (OS) and (**B**) Disease-Free Survival (DFS) in 200 patients with AML stratified by mtDNA ETC gene mutation status. (**C**) Overall Survival and (**D**) Disease-Free Survival in 180 patients with AML (excluding M3 FAB classification, PML-RARA patients) stratified by mtDNA ETC gene mutation status.

Patients with mtDNA ETC gene mutations had significantly shorter median overall survival than patients without mtDNA ETC mutations (median: 9.3 vs 20.1 months; p-value: 0.007; log-rank (Mantel-Cox) test, Fig. 2A). No significant difference in disease-free survival was detected between the ETC mutant and wild type patients (Fig. 2B). Because patients with APL or t(15;17) receive different treatment (ATRA) than other patients with AML and have better clinical outcome, we excluded these patients and reanalyzed the association between the presence of mtDNA ETC mutations and patients’ survival. Similarly, patients with mtDNA ETC gene mutations had shorter overall survival (median: 9.3 vs 15.8; p-value: 0.014) but not disease-free survival (Fig. 2C,D). After age-stratification, however, the difference in median overall survival was lost, likely due to the small number of patients (Supplemental Fig. S1).

In multivariable survival analysis using the Cox Proportional Hazards model—after adjustment for age, cytogenetic risk, transplant status and mutations in *DNMT3A*, *RUNX1* and *TP53*—the association between ETC mutations and clinical outcome was not statistically significant (p-value: 0.229; Table 4).

**Table 4:** Cox Proportional Hazards modeling for overall survival in patients with mutations in the mtDNA ETC genes (n = 194).

Variables	Hazard Ratio	95% CI		p-value
Age	1.02	1.00	1.03	0.037
Cytogenetic Risk				
*Intermediate*	3.22	1.65	6.28	0.001
*Poor*	5.35	2.42	11.8	<0.001
Transplant Status	0.44	0.29	0.68	<0.001
*DNMT3A*	1.47	0.96	2.25	0.080
*RUNX1*	1.82	0.96	3.44	0.066
*TP53*	2.00	1.02	3.94	0.044
*mtDNA ETC*	1.44	0.79	2.62	0.229

**Figure 3:**
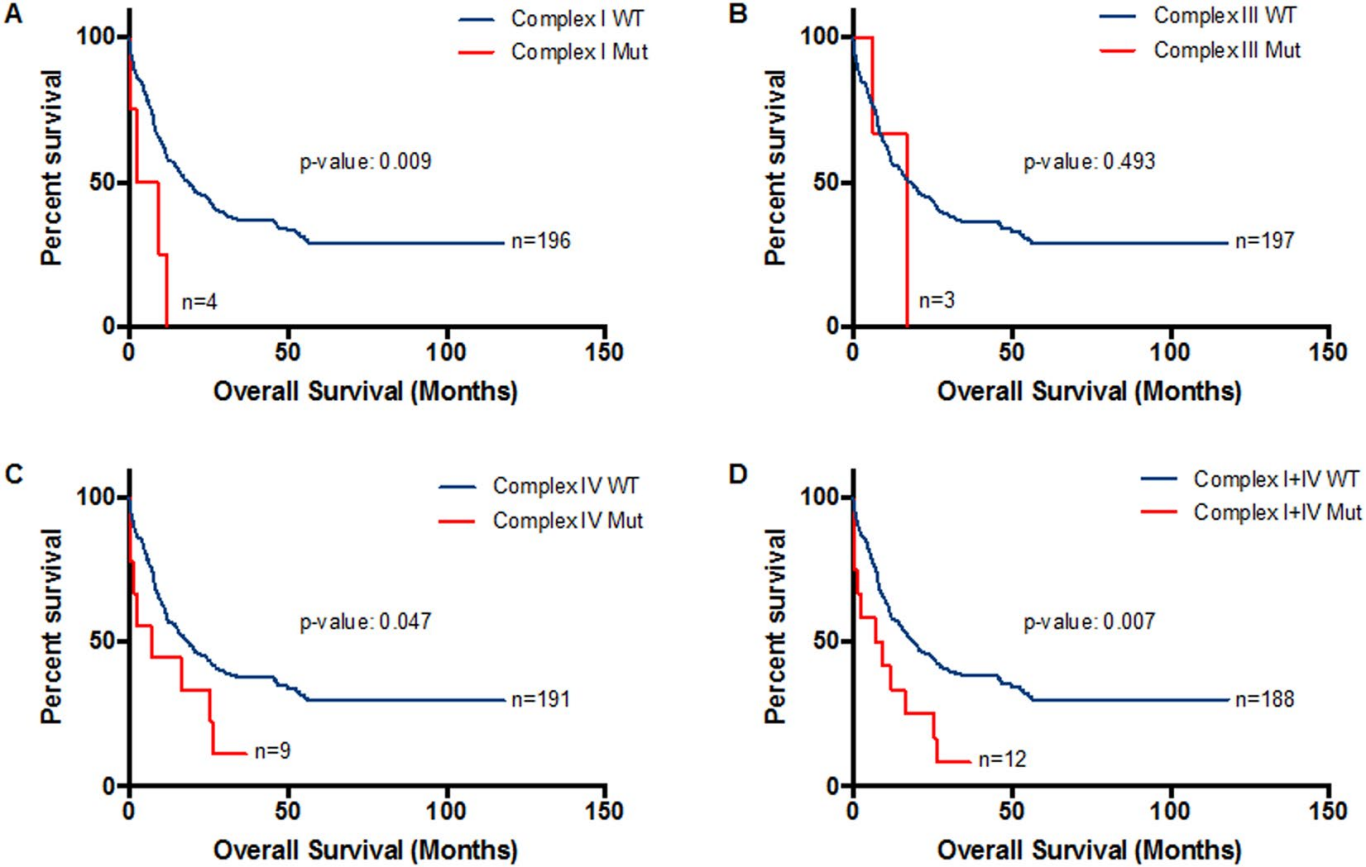
Kaplan-Meier survival curves for Complex I, III and IV of ETC genes in AML. (**A**) OS in patients with (n = 4) and without (n = 196) mutations in Complex I. (**B**) OS in patients with (n = 3) and without (n = 197) mutations in Complex III. (**C**) OS in patients with (n = 8) and without (n = 192) mutations in Complex IV. (**D**) OS in patients with (n = 12) and without (n = 188) mutations in either or both Complex I and IV.

Patients with mutations in Complex I had a significantly shorter median overall survival than patients without mutations (5.85 vs 18.5 months; p-value: 0.009; Fig. 3A) compared with patients without the mutations. Patients with mutations in Complex III did not have a significant difference in overall survival (Fig. 3B). Patients with mutations in Complex IV had significantly shorter median overall survival than patients without mutations (median: 7.0 vs 18.5 months; p-value: 0.047; Fig. 3C). Thus, we performed survival analysis combining the presence of mutations in either or both Complex I and Complex IV. Patients with mutations in either or both Complex I and IV had significantly poorer overall survival than patients without mutations (median: 8.15 vs 19 months; p-value: 0.007; Fig. 3D).

**Figure 4:**
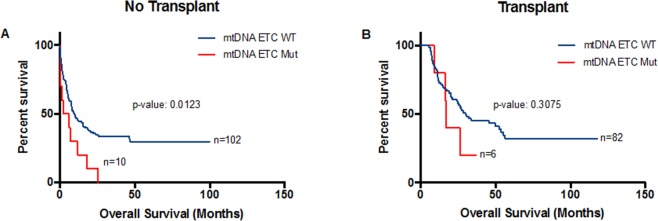
Kaplan-Meier survival curves for patients with AML according to ETC gene mutations after stratification by whether patients received transplant or not. Overall survival of patients who (**A**) did not receive transplant or (**B**) did receive transplant stratified by mitochondrial ETC gene mutation status.

We also stratified patients by whether they received transplant or not. We found a significant association between mtDNA ETC gene mutations and poorer overall survival in patients who did not receive transplant (median: 4.35 vs 9.9 months; p-value: 0.0123; Fig. 4A). In patients who received transplant, there was also a trend towards worse overall survival in patients with mtDNA ETC gene mutations compared with those without mutations but this trend did not reach statistical significance (Fig. 4B).

**Figure 5:**
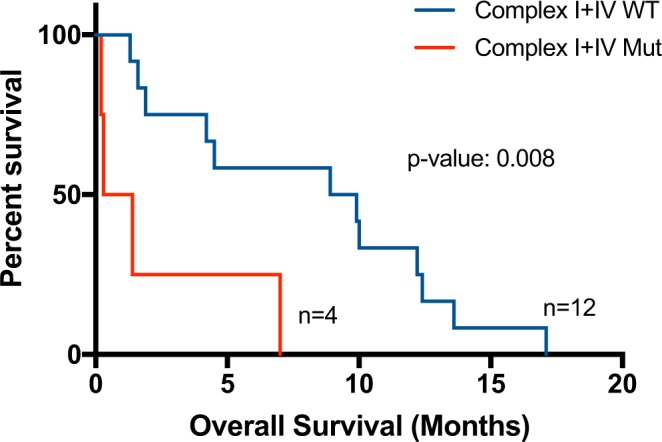
Kaplan-Meier survival curves of patients with AML according to mutations in either or both Complex I and IV of ETC genes in *TP53* mutation positive patients. OS in *TP53* mutated patients who had (n = 4) or did not have (n = 12) mutations in either or both Complex I and IV.

Because mutations in either or both Complex I and IV were associated with *TP53* mutation, we also stratified patients according to *TP53* mutational status. We found that among *TP53*-mutant patients, those with mutations in either or both Complex I and IV had significantly worse overall survival than those without mutations (median: 0.85 vs 9.4 months; p-value: 0.008; Fig. 5).

Using the Cox Proportional Hazards model we adjusted for age, cytogenetic risk, transplant status, *DNMT3A*, *RUNX1* and *TP53* mutations and found that in survival analysis, mutations in Complex I, alone, Complex III, alone, Complex IV, alone or either or both Complex I and IV were not significantly associated with overall survival.

## Discussion

Acute myeloid leukemic cells are highly oxidized when compared with healthy hematopoietic stem cells; thus, mechanisms that contribute to this oxidation state represent an opportunity for targeted therapy^14,15,21^. Mutations in the mtDNA have been reported in several malignancies including AML^20,22–24^; the majority of these studies examined a limited number of patients and focused on targeted sequencing analysis. Thus, characterization of mtDNA mutations particularly those in genes involved in OXPHOS in a larger AML cohort is needed. Using the TCGA dataset, we identified 16 of 200 (8%) patients with AML had a mutation in one or more of their mitochondrial genes encoding ETC complexes. The high frequency of mtDNA ETC mutations in AML is similar to or greater than that of *NRAS (8%)*, *WT1 (6%)*, *CEBPA (7%)* and *TP53 (8%)* mutations—a few of the top 12 genes that are regularly mutated in AML, thus implicating these mutations in AML pathogenesis.

Complex I, or NADH dehydrogenase, binds NADH and transfers two electrons to ubiquinone (UQ) generating NAD^+^ and ubiquinol (UQH_2_) and pumps four H^+^ into the inner membrane space. Complex II, or succinate dehydrogenase, oxidizes succinate and transfers two electrons to quinone via FAD; electrons can also enter the ETC from this complex but this complex does not directly contribute to the proton gradient. Complex III, or cytochrome c oxidoreductase, pumps two H+ ions into the inner membrane space by oxidizing ubiquinone to ubiquinol^25^. Then the electrons are transferred through cytochrome bc_1_ to cytochrome c, which carries the electrons to complex IV^25^. At Complex IV, or cytochrome c oxidase, electrons carried over from cytochrome c are used to reduce molecular oxygen to two water molecules. Although mtDNA mutations were present in all complexes of the ETC, the majority of the mutations were found in Complex IV. Mutations in *MT-CO1* and *MT-CO2* of Complex IV have been previously reported in AML and were found to be associated with inferior disease-free survival^23^. Complex IV produces H_2_O from O_2_ and contributes protons to the proton gradient. The second most frequently mutated complex is Complex I, which is a major site of electron leakage that leads to the formation of superoxide anions. A previous study reported a potential association between the presence of mtDNA mutation in the *ND1* gene of Complex I and the abnormal ROS production in blasts obtained from one patient with megacaryoblastic leukaemia^26^. Patients with mutations in either or both of these complexes had worse overall survival than patients without mutations in these complexes. While Complex III is also involved in electron transport and prone to electron leakage, its contribution to the production of superoxide is limited^27,28^. Nevertheless, the lack of association between mutations in Complex III and clinical outcome is likely due to the small number of patients carrying these mutations.

The role of mitochondrial dysfunction in aging is supported by several lines of evidence. Furthermore, previous studies have shown that somatic variation in mitochondrial DNA occur more frequently in aging tissues^29^. Our study further demonstrated the association between mutations in the mtDNA of ETC genes with age. This is also important since older patients with AML have significantly poorer clinical outcome compared with younger patients, and less than 10% of older patients achieve long term survival. Because ETC mutations occur more frequently in older patients and are potentially associated with worse survival, they present an opportunity for biomarker and drug development. Compared with normal hematopoietic cells, AML cells have greater mitochondrial mass without increasing respiratory chain complex activity. The lower spare reserve capacity in the respiratory chain makes it more susceptible to oxidative stress. Therefore, strategies that target the ETC chain may be effective against AML cells^16^.

The association between mitochondrial mutations in the ETC and mutations in *TP53* is particularly interesting. p53 is known to translocate to the mitochondria during stress to activate apoptotic pathways^30^. p53 is also involved in regulating cellular respiration and energy metabolism, p53 has been found to induce expression and synthesis of cytochrome c oxidase (Complex IV)^31,32^ and possibly contributes to the Warburg effect. The Electron Transport Chain is a critical generator of reactive oxygen species in the mitochondria, and ROS has been shown to affect p53 stress sensitivity in leukemic cells^33^. A previous study demonstrated that translocation of p53 to the mitochondria to initiate apoptosis leads to decreased SOD2 activity and increased superoxide content^34^. Additionally, another study indicated an important role of p53 in maintaining genomic stability of mtDNA: via direct interaction between p53 and mtDNA polymerase γ. Conversely, loss of p53 can lead to increased susceptibility of mtDNA to mutations^35^. This could possibly explain the association between mtDNA ETC gene mutations and *TP53* mutations. However, further mechanistic studies are needed to elucidate the exact relationship between *TP53* mutations and mtDNA ETC gene mutations. Furthermore, patients with *TP53* mutations are known to have inferior outcome in AML^36^. Here, we showed that further stratification of *TP53* mutation positive patients by mutation status in either or both Complex I and IV was associated with even worse survival.

One limitation of this analysis, particularly those related to the association with clinical outcome would be the relatively small sample size of patients with mutations in each complex compared to patients without any mutation, especially after age-stratification. However, since this study analyzed the largest publically available dataset of AML, this triggers the need for including mtDNA in the mutational analysis of patients with AML.

Mitochondrial mutations have previously been proven to drive cancer pathogenesis in other cancers, thus expanding the research to study their role more critically in AML pathogenesis is of great interest. Here, we characterize mutations in mtDNA ETC genes in AML, we report their frequencies and association with age, *TP53* mutations and patients’ clinical outcome. Future studies into the role of mitochondrial mutations in AML could further elucidate the mechanisms by which AML pathogenesis and progression occurs, as well as possibly identify new therapeutic targets.

## Methods

### Patient Data from The Cancer Genome Atlas (TCGA) dataset

Molecular and clinical patient data used for this analysis were retrieved from the Cancer Genome Atlas (TCGA) dataset from cBioPortal^37,38^, where scientists examined tumor specimens from 200 patients with AML^39^. Patients analyzed in the publically available TCGA dataset were enrolled in a single-institution tissue-banking protocol at Washington University. Patients from the provisional TCGA dataset with complete clinical (including National Comprehensive Cancer Network (NCCN) cytogenetic risk groups) and somatic mutation were included in the present analysis. We included 200 patients in our analysis for mitochondrial mutation status, clinical and molecular characteristics, as well as clinical outcome. These patient data were derived from an already published, publically available dataset and analyzed, thus ethical approval was not needed.

### Statistical Analysis

Overall Survival was defined as time between initial diagnosis and death from any cause; Event-Free Survival was defined as time between initial diagnosis and withdrawal from study due to lack of complete remission, relapse or death. Survival curves were generated using the Kaplan-Meier survival curve between patients with and without mutations in their Electron Transport Chain complexes and ATP Synthase to delineate differences in survival due to the presence of mutations. Fisher Exact, for categorical variables, and the Mann-Whitney U test, for continuous variables, were used to assess the association between the presence of ETC mutations and clinical and molecular characteristics. ETC mutations were stratified into six groups: patients with mutations in any ETC complex, patients with mutations in Complex I, patients with mutations in Complex III, patients with mutations in Complex IV, patients with mutations in either or both Complex I and IV and patients with no mutations. Of the 13 ETC transport genes encoded in the mitochondrial genome, genes for Complex II are not mitochondrial-encoded, and thus any potential mutations that exist in Complex II were not included in these analyses. The Cox Proportional Hazards model was used to assess the association between various risk factors such as mitochondrial DNA mutation status, age, cytogenetic risk, etc. and overall and disease-free survival. We used a statistical cut-off of p-value ≤ 0.05 for inclusion of variables from univariate analysis to multivariate analysis. All statistical analyses were conducted using STATA 15.1 SE and GraphPad Prism.

## Electronic supplementary material

Supplemental Material
