# Supplementary material for: Characterization of Mutations in the Mitochondrial Encoded Electron Transport Chain Complexes in Acute Myeloid Leukemia
Source: Sci Rep. 2018 Sep 5;8:13301. doi: 10.1038/s41598-018-31489-0 (PMC6125587; doi:10.1038/s41598-018-31489-0)
Supplement: Supplementary file 1 — Supplemental Material [file 41598_2018_31489_MOESM1_ESM.pdf]

Characterization of Mutations in the Mitochondrial Encoded Electron Transport Chain Complexes in Acute Myeloid Leukemia

Sharon Wu[1], Mojtaba Akhtari[2], Houda Alachkar*[1,2]

[1]USC School of Pharmacy, University of Southern California, Los Angeles, CA

[2]Norris Comprehensive Cancer Center, University of Southern California, Los Angeles, CA

Table S1. Clinical characteristics of patients with AML in the TCGA data set with available information on Complex I Mutations. *P-values calculated using non-parametric Mann-Whitney U or Fisher's Exact test. Two patients were missing FAB classification data.*

Characteristic	No Mutations (n=196)	Mutations (n=4)	*p-value*
**Age, median** (years)	57	68	0.15
Young (<60)	108 (55.1%)	1 (25%)	0.33
Old (≥60)	88 (44.9%)	3 (75%)	
**Sex**			0.63
Female *(n, %)*	91 (46.4%)	1 (25%)	
Male *(n, %)*	105 (53.6%)	3 (75%)	
**FAB**			
M0 (n, %)	19 (9.74%)	0	>0.99
M1 *(n, %)*	45 (23.1%)	1 (33.3%)	0.55
M2 *(n, %)*	43 (22.1%)	1 (33.3%)	0.53
M3 *(n, %)*	20 (10.3%)	0	>0.99
M4 *(n, %)*	40 (20.5%)	1 (33.3%)	0.50
M5 *(n, %)*	22 (11.3%)	0	>0.99
M6 *(n, %)*	3 (1.54%)	0	>0.99
M7 *(n, %)*	3 (1.54%)	0	>0.99
**WB Count, median**	15.6	37.9	0.55
ln (WB Count)	2.60	3.08	0.56
**% BM Blast, median**	73	72.5	0.97
**% PB Blast, median**	35	26	0.45
**Risk Status**			
Poor (n, %)	49 (25%)	2 (50%)	0.28
Intermediate (n, %)	104 (53.1%)	2 (50%)	>0.99
Good (n, %)	39 (19.9%)	0	0.59
**Cytogenetic Status**			>0.99
Normal (n, %)	90 (47.1%)	2 (50%)	
Abnormal (n, %)	101 (52.9%)	2 (50%)	
**Transplant (Y/N)**			0.63
No (n, %)	109 (55.6%)	3 (75%)	
Yes (n, %)	87 (44.4%)	1 (25%)	

Table S2. Clinical characteristics of patients with AML in the TCGA data set with available information on Complex III Mutations. *P-values calculated using non-parametric Mann-Whitney U or Fisher's Exact test.*

Characteristic	No Mutations (n=197)	Mutations (n=3)	*p-value*
**Age, median** (years)	57	65	0.14
Young (<60)	109 (55.3%)	0	0.09
Old (≥60)	88 (44.7%)	3 (100%)	
**Sex**			0.25
Female *(n, %)*	92 (46.7%)	0	
Male *(n, %)*	105 (53.3%)	3 (100%)	
**FAB**			
M0 (n, %)	18 (9.23%)	1 (33.3%)	0.26
M1 *(n, %)*	44 (22.6%)	2 (66.7%)	0.14
M2 *(n, %)*	44 (22.6%)	0	>0.99
M3 *(n, %)*	20 (10.3%)	0	>0.99
M4 *(n, %)*	41 (21.0%)	0	>0.99
M5 *(n, %)*	22 (11.3%)	0	>0.99
M6 *(n, %)*	3 (1.54%)	0	>0.99
M7 *(n, %)*	3 (1.54%)	0	>0.99
**WB Count, median**	16	48	0.84
ln (WB Count)	2.61	2.66	0.96
**% BM Blast, median**	72	83	0.30
**% PB Blast, median**	34.5	49	0.91
**Risk Status**			
Poor (n, %)	50 (25.4%)	1 (33.3%)	>0.99
Intermediate (n, %)	104 (52.8%)	2 (66.7%)	>0.99
Good (n, %)	39 (19.8%)	0	>0.99
**Cytogenetic Status**			0.60
Normal (n, %)	90 (46.9%)	2 (66.7%)	
Abnormal (n, %)	102 (53.1%)	1 (33.3%)	
**Transplant (Y/N)**			0.58
No (n, %)	111 (56.3%)	1 (33.3%)	
Yes (n, %)	86 (43.7%)	2 (66.7%)	

Table S3. Clinical characteristics of patients with AML in the TCGA data set with available information on Complex IV Mutations. *P-values calculated using non-parametric Mann-Whitney U or Fisher's Exact test.*

Characteristic	No Mutations (n=191)	Mutations (n=9)	*p-value*
**Age, median** (years)	57	72	0.009
Young (<60)	107 (56%)	2 (22.2%)	0.08
Old (≥60)	84 (44%)	7 (77.8%)	
**Sex**			
Female *(n, %)*	87 (45.6%)	5 (55.6%)	0.74
Male *(n, %)*	104 (54.5%)	4 (44.4%)	
**FAB**			
M0 (n, %)	16 (8.47%)	3 (33.3%)	0.04
M1 *(n, %)*	43 (22.8%)	3 (33.3%)	0.44
M2 *(n, %)*	42 (22.2%)	2 (22.2%)	>0.99
M3 *(n, %)*	19 (10.1%)	1 (11.1%)	>0.99
M4 *(n, %)*	41 (21.7%)	0	0.21
M5 *(n, %)*	22 (11.6%)	0	0.60
M6 *(n, %)*	3 (1.59%)	0	>0.99
M7 *(n, %)*	3 (1.59%)	0	>0.99
**WB Count, median**	16.8	5.4	0.56
ln (WB Count)	2.63	2.37	0.63
**% BM Blast, median**	73	74	0.98
**% PB Blast, median**	35	36	0.76
**Risk Status**			
Poor (n, %)	46 (24.1%)	5 (55.6%)	0.053
Intermediate (n, %)	103 (53.9%)	3 (33.3%)	0.31
Good (n, %)	38 (19.9%)	1 (11.1%)	0.69
**Cytogenetic Status**			0.50
Normal (n, %)	89 (47.9%)	3 (33.3%)	
Abnormal (n, %)	97 (52.2%)	6 (66.7%)	
**Transplant (Y/N)**			0.73
No (n, %)	106 (55.5%)	6 (66.7%)	
Yes (n, %)	85 (44.5%)	3 (33.3%)	

Table S4. Clinical characteristics of patients with AML in the TCGA data set with available information on mutations in either Complex I or IV. *P-values calculated using non-parametric Mann-Whitney U or Fisher's Exact test.*

**Characteristic**	**No Mutations (n=188)**	**Mutations (n=12)**	***p-value***
**Age, median** (years)	57	71.5	0.008
Young (<60)	106 (56.4%)	3 (25%)	0.04
Old (≥60)	82 (43.6%)	9 (75%)	
**Sex**			>0.99
Female *(n, %)*	87 (46.3%)	5 (41.7%)	
Male *(n, %)*	101 (53.7%)	7 (58.3%)	
**FAB**			
M0 (n, %)	16 (8.56%)	3 (27.3%)	0.08
M1 *(n, %)*	43 (23%)	3 (27.3%)	0.72
M2 *(n, %)*	41 (21.9%)	3 (27.3%)	0.71
M3 *(n, %)*	19 (10.2%)	1 (9.09%)	>0.99
M4 *(n, %)*	40 (21.4%)	1 (9.09%)	0.47
M5 *(n, %)*	22 (11.8%)	0	0.62
M6 *(n, %)*	3 (1.60%)	0	>0.99
M7 *(n, %)*	3 (1.60%)	0	>0.99
**WB Count, median**	16.3	14.5	0.90
ln (WB Count)	2.61	2.71	0.83
**% BM Blast, median**	72.5	74.5	0.67
**% PB Blast, median**	34.5	52	0.80
**Risk Status**			
Poor (n, %)	44 (23.4%)	7 (58.3%)	0.015
Intermediate (n, %)	102 (54.2%)	4 (33.33%)	0.15
Good (n, %)	38 (20.2%)	1 (8.33%)	0.47
**Cytogenetic Status**			0.38
Normal (n, %)	88 (48.1%)	4 (33.3%)	
Abnormal (n, %)	95 (51.9%)	8 (66.7%)	
**Transplant (Y/N)**			0.56
No (n, %)	104 (55.3%)	8 (66.7%)	
Yes (n, %)	84 (44.7%)	4 (33.3%)	

Table S5. Molecular characteristics of patients with AML in the TCGA data set with available information on mutation status in Complex IV. *P-values calculated using non-parametric Mann-Whitney U or Fisher's Exact test.*

Gene	No Mutations (n=191)	Mutations (n=9)	*p-value*
FLT3	55 (28.8%)	1 (11.1%)	0.45
TP53	13 (6.81%)	3 (33.3%)	0.026
DNMT3A	47 (24.6%)	2 (22.2%)	>0.99
CEBPA	12 (6.28%)	1 (11.1%)	0.46
NRAS	15 (7.85%)	0	>0.99
TET2	16 (8.38%)	1 (11.1%)	0.56
IDH1	19 (9.95%)	0	>0.99
IDH2	20 (10.5%)	0	0.60
RUNX1	16 (8.38%)	1 (11.1%)	0.56
NPM1	53 (27.7%)	1 (11.1%)	0.45
WT1	12 (6.28%)	0	>0.99

Table S6. Molecular characteristics of patients with AML in the TCGA data set with available information on mutations in either or both Complex I and IV. *P-values calculated using non-parametric Mann-Whitney U or Fisher's Exact test.*

Gene	No Mutations (n=188)	Mutations (n=12)	*p-value*
FLT3	55 (29.3%)	1 (8.33%)	0.19
TP53	12 (6.38%)	4 (33.3%)	0.009
DNMT3A	46 (24.5%)	3 (25%)	>0.99
CEBPA	12 (6.38%)	1 (8.33%)	0.56
NRAS	15 (7.98%)	0	0.61
TET2	16 (8.51%)	1 (8.33%)	>0.99
IDH1	18 (13.3%)	1 (8.33%)	>0.99
IDH2	20 (13.8%)	0	0.61
RUNX1	16 (8.51%)	1 (8.33%)	>0.99
NPM1	52 (27.7%)	2 (16.7%)	0.52
WT1	12 (6.38%)	0	>0.99

Table S7. Molecular characteristics of patients with AML in the TCGA data set with available information on mutation status in Complex I. *P-values calculated using non-parametric Mann-Whitney U or Fisher's Exact test.*

**Gene**	**No Mutations (n=196)**	**Mutations (n=4)**	***p-value***
FLT3	56 (28.6%)	0	0.58
TP53	15 (7.65%)	1 (25%)	0.29
DNMT3A	48 (24.5%)	1	>0.99
CEBPA	13 (6.63%)	0	>0.99
NRAS	15 (7.65%)	0	>0.99
TET2	17 (8.67%)	0	>0.99
IDH1	18 (12.8%)	1 (25%)	0.33
IDH2	20 (13.8%)	0	>0.99
RUNX1	16 (8.16%)	1 (25%)	0.30
NPM1	53 (27.0%)	1 (25%)	>0.99
WT1	12 (6.12%)	0	>0.99

Table S8. Molecular characteristics of patients with AML in the TCGA data set with available information on mutation status in Complex III. *P-values calculated using non-parametric Mann-Whitney U or Fisher's Exact test.*

Gene	No Mutations (n=197)	Mutations (n=3)	*p-value*
FLT3	54 (27.4%)	2 (66.7%)	0.19
TP53	15 (7.61%)	1 (33.3%)	0.22
DNMT3A	48 (24.4%)	1 (33.3%)	0.57
CEBPA	12 (6.09%)	1 (33.3%)	0.18
NRAS	15 (7.61%)	0	>0.99
TET2	17 (8.63%)	0	>0.99
IDH1	19 (9.64%)	0	>0.99
IDH2	19 (9.64%)	1 (33.3%)	0.27
RUNX1	17 (8.63%)	0	>0.99
NPM1	53 (26.9%)	1 (33.3%)	>0.99
WT1	12 (6.09%)	0	>0.99

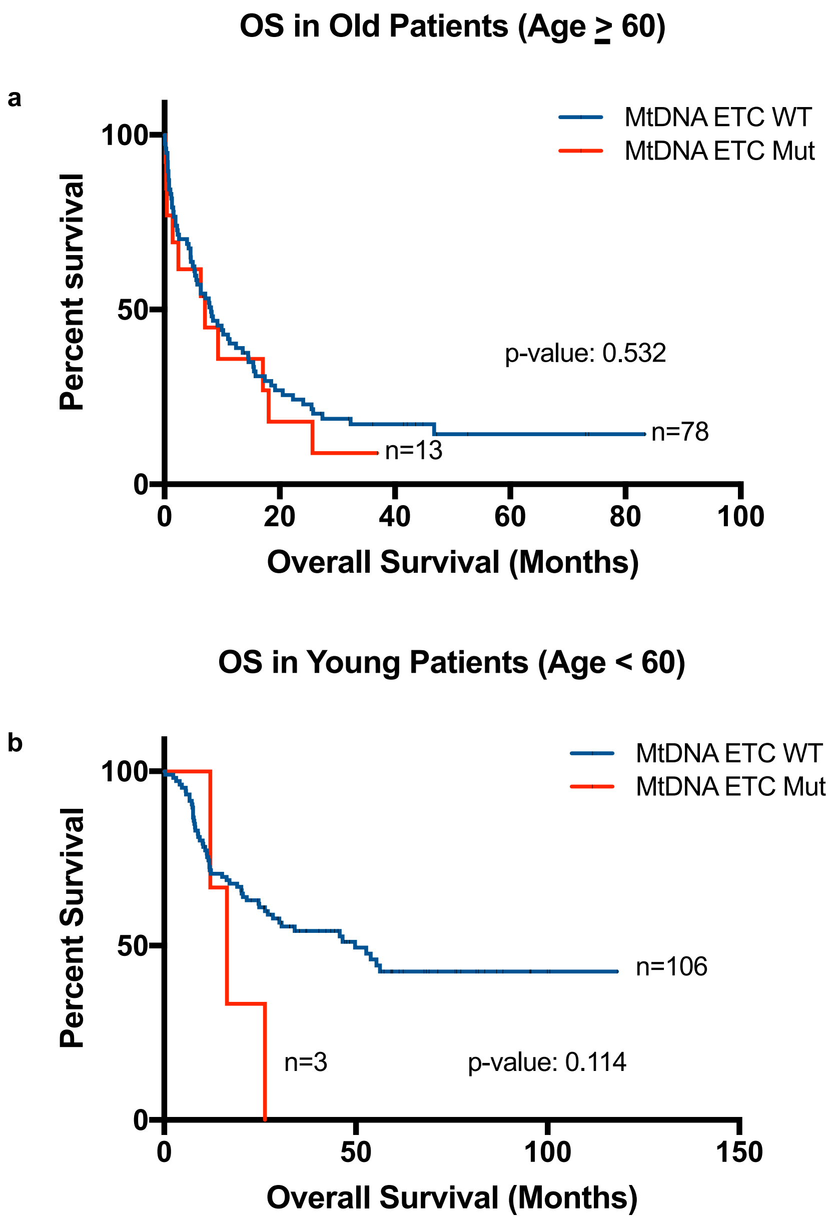

Fig S1. Kaplan-Meier Survival Curves for ETC gene mutations in AML after age-stratification. Overall survival in (a) old (age ≥ 60) and (b) young (age < 60) patients with and without mitochondrial ETC gene mutations.
